# Supplementary material for: Proteomics-driven noninvasive screening of circulating serum protein panels for the early diagnosis of hepatocellular carcinoma
Source: Nat Commun. 2023 Dec 18;14:8392. doi: 10.1038/s41467-023-44255-2 (PMC10728065; doi:10.1038/s41467-023-44255-2)
Supplement: Supplementary file 1 — Supplementary information [file 41467_2023_44255_MOESM1_ESM.pdf]

Supplementary information

Proteomics-driven noninvasive screening of circulating serum protein panels for the early diagnosis of hepatocellular carcinoma

Xing *et al.*

Supplementary Table. 1 | List of 11 peptides targeted in the PRM analysis.

Peptides	Sequence	Corresponding protein	m/z	Charge (z)	Probability	RT (min)
Peptide 1	DSDLLSPSDFK	MASP1	612.294	2	100%	55.7576
Peptide 2	SSDANLYR	CAMP	463.222	2	100%	11.9008
Peptide 3	DLHPNTDPFK	CDH13	592.291	2	100%	32.7879
Peptide 4	RPGVYTQVTK	HABP2	574.825	2	100%	13.3093
Peptide 5	NDGIIYR	ADGRG6	425.724	2	100%	19.6331
Peptide 6	ASYPDITGEK	CETP	540.765	2	100%	25.2126
Peptide 7	TSYQVYSK	CD163	488.244	2	100%	13.7488
Peptide 8	FQASVATPR	MBL2	488.764	2	100%	22.4555
Peptide 9	IETTPESR	VCAM1	466.738	2	100%	9.7477
Peptide 10	VQLYDLGLQIHK	LBP	713.906	2	100%	58.2628
Peptide 11	VGLSDAFVVVHR	PLXDC2	433.578	3	100%	57.6918
# The retention time of peptide was extracted from a 70 min gradient in shotgun proteomics analysis						

Supplementary Table. 2 | Performance of 5 potential biomarkers and different combinations of permutations in the validation set between HCC and LC patients.

Panel	Sensitivity	Specificity	AUC (95%CI)
HABP2+CD163	0.838	0.872	0.935 (0.89-0.98)
HABP2+CD163+MBL2+CAMP	0.925	0.738	0.932 (0.89-0.98)
HABP2+CD163+CAMP	0.900	0.804	0.930 (0.89-0.97)
HABP2+CD163+MBL2+CETP+CAMP	0.913	0.718	0.930 (0.89-0.97)
HABP2+CAMP	0.763	0.935	0.925 (0.87-0.98)
HABP2+CD163+MBL2+CETP	0.875	0.869	0.925 (0.88-0.97)
HABP2+MBL2+CAMP	0.975	0.674	0.925 (0.88-0.97)
HABP2+MBL2+CETP+CAMP	0.963	0.738	0.925 (0.88-0.97)
HABP2+CD163+CETP	0.850	0.891	0.922 (0.87-0.97)
HABP2+CD163+MBL2	0.875	0.848	0.921 (0.87-0.97)
HABP2+CETP+CAMP	0.800	0.913	0.921 (0.87-0.97)
HABP2+MBL2+CETP	0.975	0.696	0.912 (0.86-0.97)
HABP2+MBL2	0.900	0.760	0.909 (0.86-0.96)
HBAP2	0.825	0.935	0.902 (0.84-0.96)
HABP2+CETP	0.925	0.696	0.901 (0.84-0.96)
CD163+CAMP	0.713	0.652	0.727 (0.64-0.82)
CD163+CETP	0.725	0.652	0.726 (0.64-0.82)
CD163	0.738	0.522	0.710 (0.62-0.80)
CD163+MBL2+CETP+CAMP	0.363	0.956	0.691 (0.60-0.78)
MBL2+CETP+CAMP	0.588	0.674	0.672 (0.58-0.77)
MBL2+CETP	0.700	0.522	0.665 (0.57-0.76)
CD163+MBL2+CETP	0.488	0.760	0.662 (0.57-0.72)
CD163+MBL2+CAMP	0.713	0.565	0.654 (0.55-0.75)
CAMP	0.200	0.891	0.650 (0.55-0.75)
CETP+CAMP	0.388	0.782	0.647 (0.55-0.75)
MBL2+CAMP	0.538	0.674	0.607 (0.51-0.71)
CETP	0.738	0.391	0.606 (0.50-0.71)
CD163+MBL2	0.813	0.347	0.601 (0.50-0.71)
MBL2	0.950	0.044	0.515 (0.41-0.62)

Supplementary Table. 3 | Performance of P4 panel for different clinical stages in the validation set between HCC and LC patients.

Clinical stages	Panels					
	HABP2+CD163+AFP+PIVKA-II			AFP+PIVKA-II		
	Sensitivity (95% CI)	Specificity (95% CI)	AUC (95% CI)	Sensitivity (95% CI)	Specificity (95% CI)	AUC (95% CI)
TNM I	0.875 (0.69-0.96)	0.915 (0.80-0.97)	0.965 (0.92-1.00)	0.750 (0.55-0.88)	0.915 (0.80-0.97)	0.889 (0.80-0.98)
TNM II	0.929 (0.81-0.98)	0.915 (0.80-0.97)	0.982 (0.96-1.00)	0.786 (0.64-0.88)	0.915 (0.80-0.97)	0.936 (0.89-0.99)
TNM III	1.000 (0.68-1.00)	0.915 (0.80-0.97)	0.992 (0.97-1.00)	0.875 (0.53-0.99)	0.915 (0.80-0.97)	0.957 (0.90-1.00)
TNM IV	1.000 (0.51-1.00)	0.915 (0.80-0.97)	0.984 (0.95-1.00)	1.000 (0.51-1.00)	0.915 (0.80-0.97)	0.984 (0.95-1.00)
BCLC 0-A	0.902 (0.80-0.95)	0.915 (0.80-0.97)	0.975 (0.95-1.00)	0.754 (0.63-0.85)	0.915 (0.80-0.97)	0.914 (0.86-0.97)
BCLC B	1.000 (0.51-1.00)	0.915 (0.80-0.97)	0.995 (0.98-1.00)	1.000 (0.51-1.00)	0.915 (0.80-0.97)	0.984 (0.95-1.00)
BCLC C	1.000 (0.77-1.00)	0.915 (0.80-0.97)	0.990 (0.97-1.00)	0.846 (0.58-0.97)	0.915 (0.80-0.97)	0.962 (0.92-1.00)
CNLC Ia	0.878 (0.74-0.95)	0.915 (0.80-0.97)	0.968 (0.94-1.00)	0.683 (0.53-0.80)	0.915 (0.80-0.97)	0.889 (0.82-0.96)
CNLC Ib	0.950 (0.73-1.00)	0.915 (0.80-0.97)	0.998 (0.97-1.00)	0.950 (0.76-1.00)	0.915 (0.80-0.97)	0.970 (0.94-1.00)
CNLC II	1.000 (0.51-1.00)	0.915 (0.80-0.97)	1.000 (1.00-1.00)	1.000 (0.51-1.00)	0.915 (0.80-0.97)	0.984 (0.95-1.00)
CNLC III	1.000 (0.77-1.00)	0.915 (0.80-0.97)	0.989 (0.97-1.00)	0.846 (0.58-0.97)	0.915 (0.80-0.97)	0.956 (0.91-1.00)

Supplementary Table. 4 | Performance of P4 model for HCC risk prediction in the prospective validation cohort

Panel	Prospective Training Set			Prospective Validation Set		
	Sensitivity	Specificity	AUC (95% CI)	Sensitivity	Specificity	AUC (95% CI)
P4	1.000	1.000	1.000 (1.00-1.00)	0.909	0.877	0.890 (0.74-1.00)
AFP	0.240	0.934	0.661 (0.54-0.78)	0.455	0.954	0.835 (0.69-0.98)
PIVKA-II	0.182	0.908	0.609 (0.50-0.72)	0.182	0.908	0.626 (0.45-0.80)
AFP+PIVKA-II	1.000	0.967	0.999 (0.99-1.00)	0.818	0.738	0.778 (0.61-0.94)
ASAP	0.545	0.877	0.665 (0.55-0.78)	0.545	0.877	0.711 (0.52-0.90)
aMAP	0.727	0.600	0.545 (0.43-0.67)	0.727	0.600	0.662 (0.47-0.85)

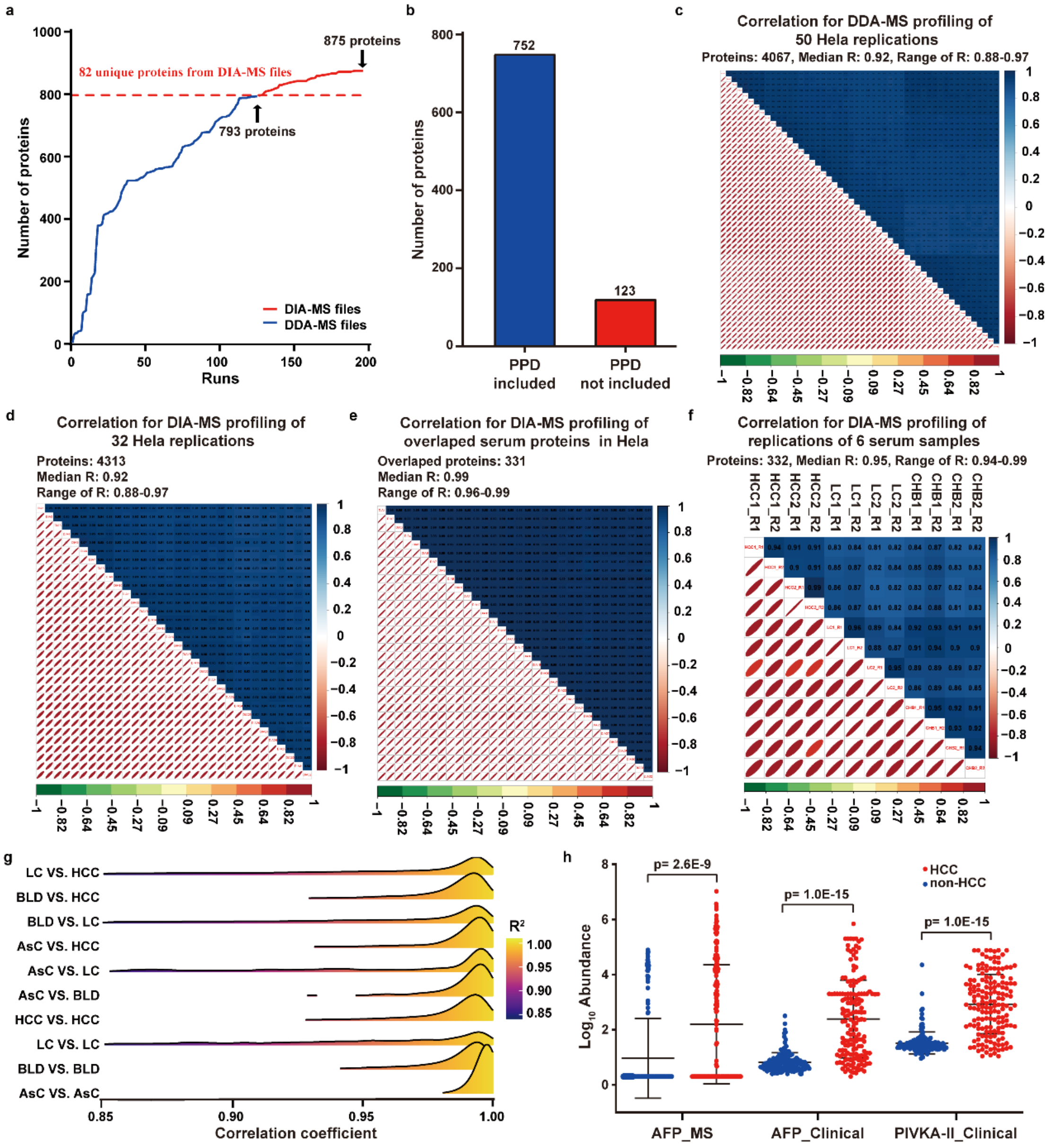

Supplementary Fig. 1 | Assessment of the mass spectrometry platform and proteomic data. a The protein number accumulation curve distinguishing the data acquisition mode. **b** Comparison of the number of proteins in the spectral library of serum samples and Plasma Proteome Database (PPD). **c** The pearson’s correlation coefficients of standards through library process in DDA mode (n = 50 independent experiments) and DIA mode (n= 32 independent experiments) (**d**). **e** The pearson’s correlation coefficients of overlapped proteins between standards and serum samples through library process in DIA mode (n = 32 independent experiments). **f** The pearson’s correlation coefficients of technical replicates of 6 serum samples through

library process in DIA mode (n = 12 independent experiments). **g** The pearson's correlation coefficient distribution of identified proteins in every two groups (AsC, n= 40 biologically independent samples; BLD, n= 64 biologically independent samples; LC, n= 53 biologically independent samples; HCC, n= 163 biologically independent samples). **h** Quantitative analysis of AFP_MS, AFP_Clinical and PIVKA-II_Clinical in HCC (n = 163 biologically independent samples) and non-HCC (n = 157 biologically independent samples) groups. Data represent mean ± SD. Significance determined by two-sided Wilcoxon test with Benjamini-Hochberg multiple test adjustment. Source data are provided as a Source Data file.

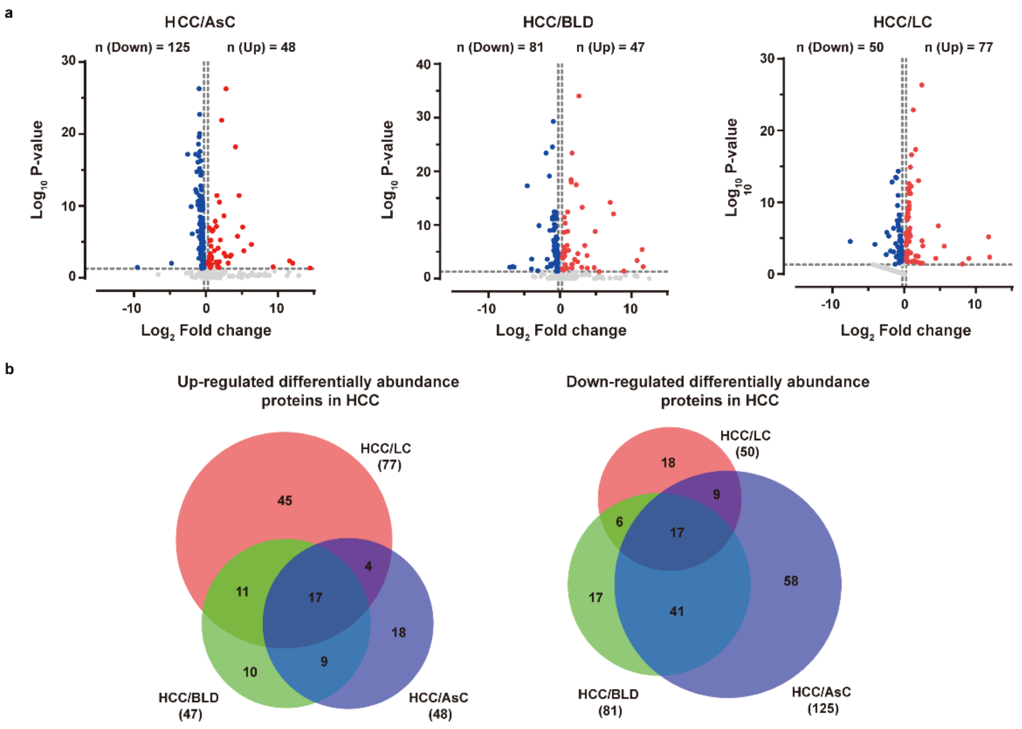

Supplementary Fig. 2 | Screening of differentially abundant proteins. a Volcano plot represented the protein abundance changes in the comparison between HCC (n = 163 biologically independent samples)/AsC (n = 40 biologically independent samples), HCC/BLD (n = 64 biologically independent samples), and HCC/LC (n = 53 biologically independent samples) groups, respectively. Dashed lines delimit significantly down-regulated (blue dots) and up-regulated proteins (red dots). **b** Venn diagrams exhibited the overlay of these differentially abundant proteins in 3 comparisons. Significance determined by two-sided independent sample t-test with Benjamini-Hochberg multiple test adjustment. Source data are provided as a Source Data file.

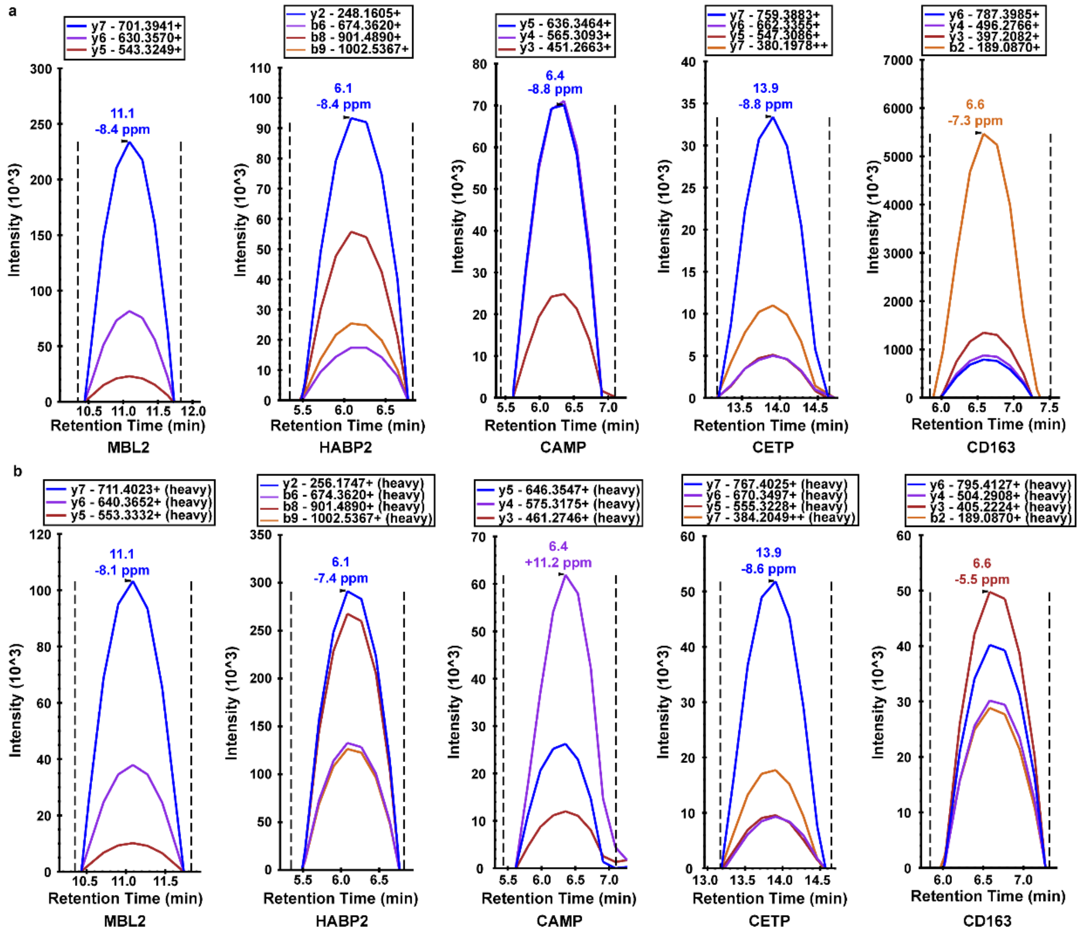

Supplementary Fig. 3 | Peak contributions of the individual fragment ions from the unique peptide of MBL2, HABP2, CAMP, CETP and CD163. a The panel showed the ions of detected peptides, **b** The panel showed the ions of the targeted peptides added with isotope. Source data are provided as a Source Data file.

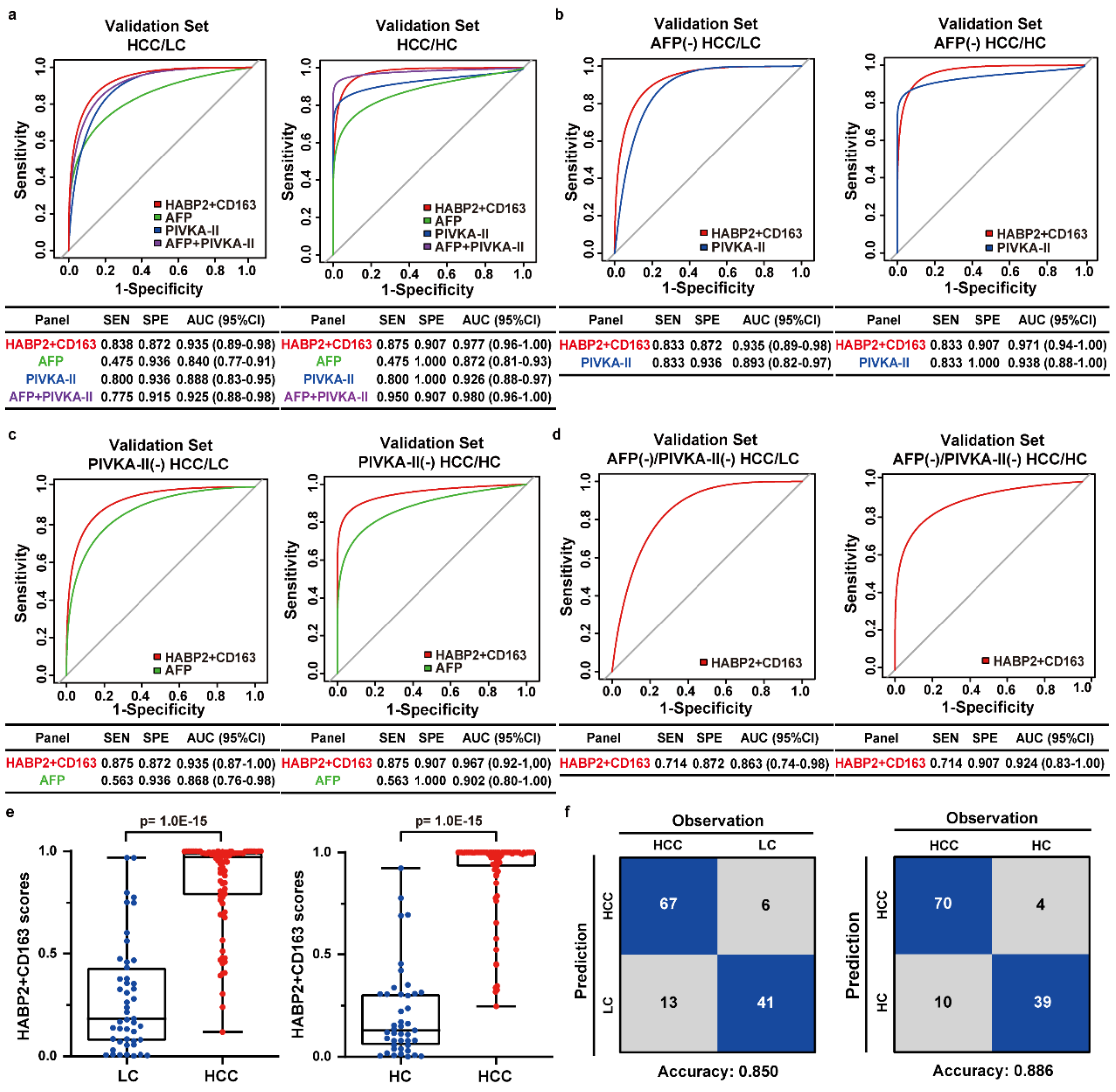

a Validation Set HCC/LC

Panel	SEN	SPE	AUC (95%CI)
HABP2+CD163	0.838	0.872	0.935 (0.89-0.98)
AFP	0.475	0.936	0.840 (0.77-0.91)
PIVKA-II	0.800	0.936	0.888 (0.83-0.95)
AFP+PIVKA-II	0.775	0.915	0.925 (0.88-0.98)

a Validation Set HCC/HC

Panel	SEN	SPE	AUC (95%CI)
HABP2+CD163	0.875	0.907	0.977 (0.96-1.00)
AFP	0.475	1.000	0.872 (0.81-0.93)
PIVKA-II	0.800	1.000	0.926 (0.88-0.97)
AFP+PIVKA-II	0.950	0.907	0.980 (0.96-1.00)

b Validation Set AFP(-) HCC/LC

Panel	SEN	SPE	AUC (95%CI)
HABP2+CD163	0.833	0.872	0.935 (0.89-0.98)
PIVKA-II	0.833	0.936	0.893 (0.82-0.97)

b Validation Set AFP(-) HCC/HC

Panel	SEN	SPE	AUC (95%CI)
HABP2+CD163	0.833	0.907	0.971 (0.94-1.00)
PIVKA-II	0.833	1.000	0.938 (0.88-1.00)

c Validation Set PIVKA-II(-) HCC/LC

Panel	SEN	SPE	AUC (95%CI)
HABP2+CD163	0.875	0.872	0.935 (0.87-1.00)
AFP	0.563	0.936	0.868 (0.76-0.98)

c Validation Set PIVKA-II(-) HCC/HC

Panel	SEN	SPE	AUC (95%CI)
HABP2+CD163	0.875	0.907	0.967 (0.92-1.00)
AFP	0.563	1.000	0.902 (0.80-1.00)

d Validation Set AFP(-)/PIVKA-II(-) HCC/LC

Panel	SEN	SPE	AUC (95%CI)
HABP2+CD163	0.714	0.872	0.863 (0.74-0.98)

d Validation Set AFP(-)/PIVKA-II(-) HCC/HC

Panel	SEN	SPE	AUC (95%CI)
HABP2+CD163	0.714	0.907	0.924 (0.83-1.00)

Supplementary Fig. 4 | Performance of the HABP2+CD163 panel in validation cohort. a ROC curves of HABP2+CD163, AFP, PIVKA-II and their combination for HCC patients (n = 80) versus LC patients (n = 47) and HCC patients (n = 80) versus HC (n = 43). **b** ROC curves of HABP2+CD163 and PIVKA-II for AFP-negative HCC patients (n = 42) versus LC patients (n = 47) and HCC patients (n = 42) versus HC (n = 43). **c** ROC curves of HABP2+CD163 and AFP for PIVKA-II -negative HCC patients (n = 16) versus LC patients (n = 47) and HCC patients (n = 16) versus HC (n = 43). **d** ROC curves of HABP2+CD163 for AFP-negative and PIVKA-II-negative HCC patients (n = 7) versus LC patients (n = 47) and HCC patients (n = 7) versus HC (n = 43). **e** Differences of HABP2+CD163 scores between HCC patients and LC patients, and HCC patients and HC. Significance was determined by two-sided

wilcoxon test with Benjamini-Hochberg multiple test adjustment. Box plots indicate median (middle line), 25%, 75% percentile (box) and minimum and maximum (whiskers) as well as outliers (single points). **f** Confusion matrix showing performance of HABP2+CD163 for classifying HCC and LC, HCC and HC in the validation set. Source data are provided as a Source Data file.

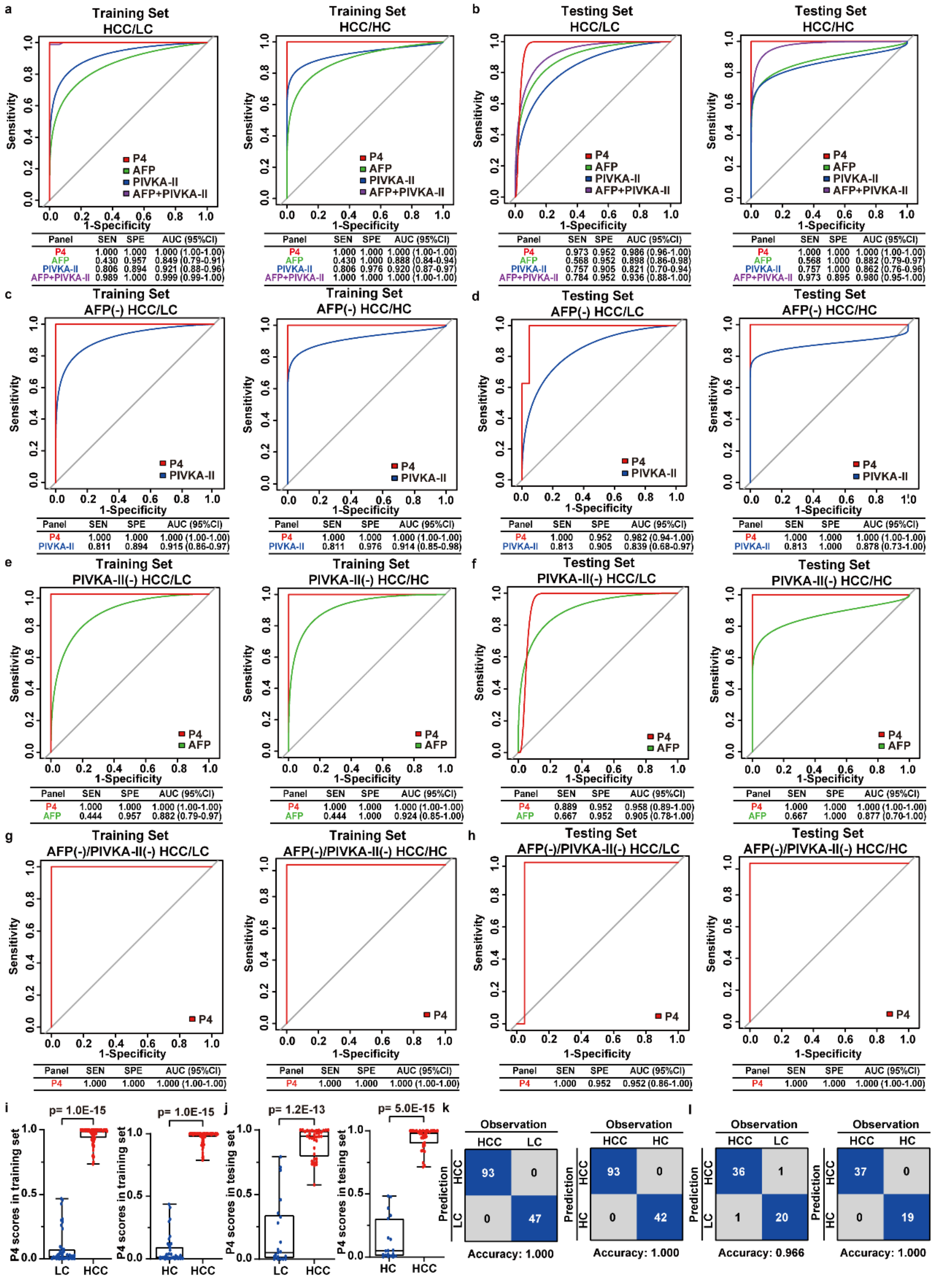

Supplementary Fig. 5 | Diagnosis performance of the P4 model in training set and testing set. a ROC curves of P4 panel, AFP, PIVKA-II and their combination for HCC patients (n = 93) versus LC patients (n = 47) and HCC patients (n = 93) versus HC (n = 42) in the training set. **b** ROC curves of P4 panel, AFP, PIVKA-II and their combination for HCC patients (n = 37) versus LC patients (n = 21) and HCC patients

(n = 37) versus HC (n = 19) in the testing set. **c** ROC curves of P4 panel and PIVKA-II for AFP-negative HCC patients (n = 53) versus LC patients (n = 47) and HCC patients (n = 53) versus HC (n = 42) in the training set. **d** ROC curves of P4 panel and PIVKA-II for AFP-negative HCC patients (n = 16) versus LC patients (n = 21) and HCC patients (n = 16) versus HC (n = 19) in the testing set. **e** ROC curves of P4 panel and AFP for PIVKA-II -negative HCC patients (n =18) versus LC patients (n = 47) and HCC patients (n = 18) versus HC (n = 42) in the training set. **f** ROC curves of P4 panel and AFP for PIVKA-II -negative HCC patients (n = 9) versus LC patients (n = 21) and HCC patients (n = 9) versus HC (n = 19) in the testing set. **g** ROC curves of P4 panel for AFP-negative and PIVKA-II-negative HCC patients (n = 10) versus LC patients (n = 47) and HCC patients (n = 10) versus HC (n = 42) in the training set. **h** ROC curves of P4 panel for AFP-negative and PIVKA-II-negative HCC patients (n = 3) versus LC patients (n = 21) and HCC patients (n = 3) versus HC (n = 19) in the testing set. **i** Differences of P4 scores between HCC patients and LC patients, and HCC patients and HC in the training set and testing set (**j**). Significance was determined by two-sided wilcoxon test with Benjamini-Hochberg multiple test adjustment. **k** Confusion matrix showing P4 panel performance for classifying HCC and LC, HCC and HC in the training set and testing set (**l**). Box plots indicate median (middle line), 25%, 75% percentile (box) and minimum and maximum (whiskers) as well as outliers (single points). Source data are provided as a Source Data file.

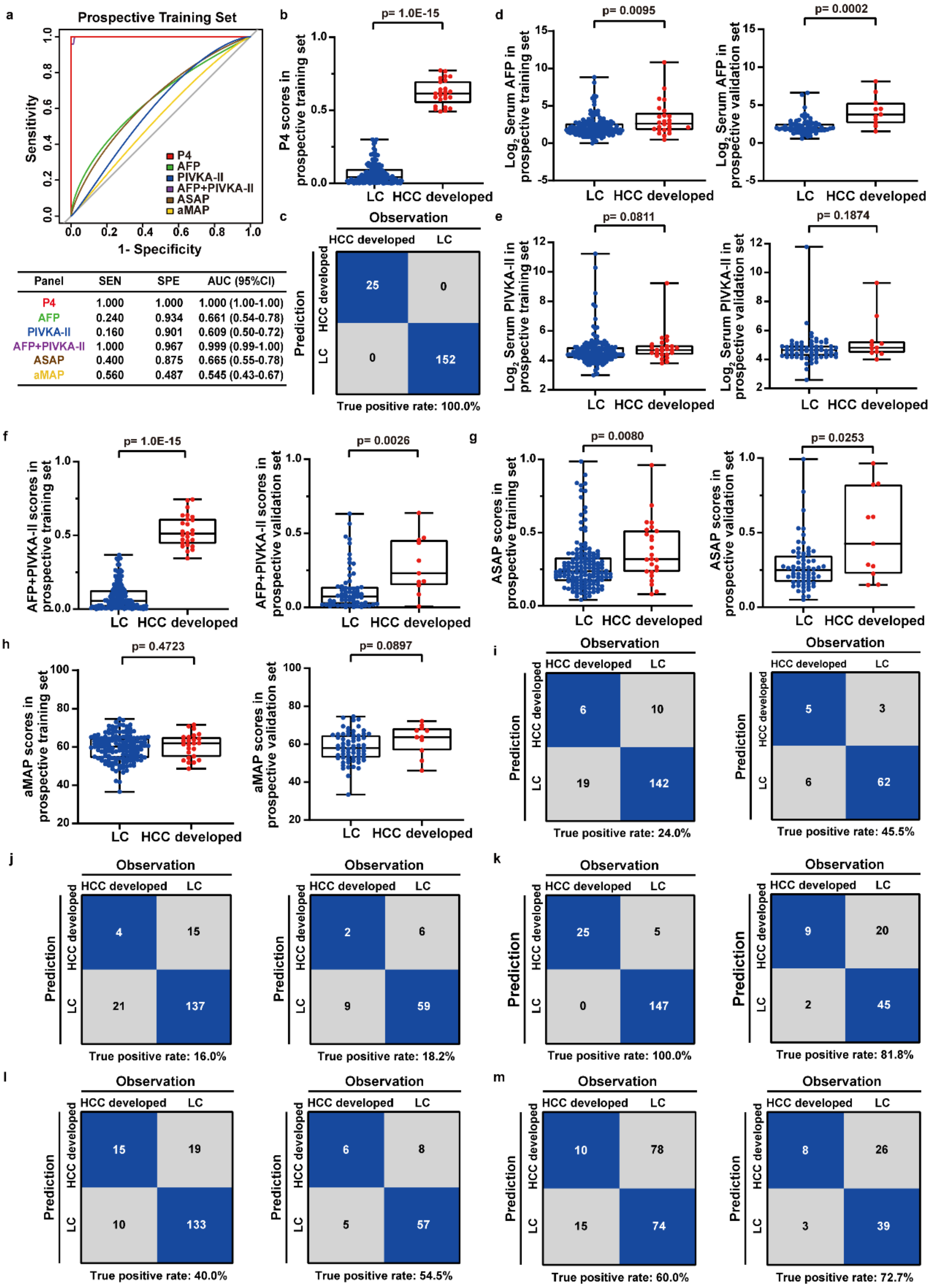

Panel	SEN	SPE	AUC (95%CI)
P4	1.000	1.000	1.000 (1.00-1.00)
AFP	0.240	0.934	0.661 (0.54-0.78)
PIVKA-II	0.160	0.901	0.609 (0.50-0.72)
AFP+PIVKA-II	1.000	0.967	0.999 (0.99-1.00)
ASAP	0.400	0.875	0.665 (0.55-0.78)
aMAP	0.560	0.487	0.545 (0.43-0.67)

Supplementary Fig. 6 | Performance of the P4 model in predicting people at high risk of HCC in the prospective training cohort. a Performance of the P4 panel, serum biomarkers (AFP, PIVKA-II, AFP+ PIVKA-II) and early diagnosis score models (ASAP and aMAP score model) for LC patients (n= 177) in prospective training cohort to predict LC patients who will develop to HCC at subsequent follow

up. The upper panel illustrated ROC curves, and the lower panel showed the AUC, sensitivity and specificity. **b** Differences of P4 scores between LC patients with and without HCC development in the training cohort. Significance was determined by two-sided wilcoxon test with Benjamini-Hochberg multiple test adjustment. **c** Confusion matrix showing P4 panel performance for classifying LC patients with and without HCC development in the training cohort. **d** Differences of serum AFP, serum PIVKA-II (**e**), AFP+PIVKA-II scores (**f**), ASAP scores (**g**) and aMAP scores (**h**) between LC patients with and without HCC developed in the training set (left) and testing set (right). Significance was determined by two-sided wilcoxon test with Benjamini-Hochberg multiple test adjustment. Box plots indicate median (middle line), 25%, 75% percentile (box) and minimum and maximum (whiskers) as well as outliers (single points). **i** Confusion matrix showing performance of serum AFP, serum PIVKA-II (**j**), AFP+PIVKA-II scores (**k**), ASAP scores (**l**) and aMAP scores (**m**) for classifying LC patients with and without HCC developed in the training set (left) and testing set (right). Source data are provided as a Source Data file.
