# Supplementary material for: Proteomics-driven noninvasive screening of circulating serum protein panels for the early diagnosis of hepatocellular carcinoma
Source: Nat Commun. 2023 Dec 18;14:8392. doi: 10.1038/s41467-023-44255-2 (PMC10728065; doi:10.1038/s41467-023-44255-2)
Supplement: Supplementary file 3 — Description of Additional Supplementary Files [file 41467_2023_44255_MOESM3_ESM.pdf]

Description of Additional Supplementary Files

Supplementary Data 1
Description: Detailed clinicopathological characteristics of 320 patients in the discovery cohort

Supplementary Data 2
Description: Statistic of clinicopathological characteristics of 320 patients in the discovery cohort

Supplementary Data 3
Description: Proteomic expression profiling of 320 samples in the discovery cohort

Supplementary Data 4
Description: List of HCC-related DEPs in serum proteomics

Supplementary Data 5
Description: Targeted proteomic expression profiles of 429 samples in the validation cohort

Supplementary Data 6
Description: Targeted proteomic expression profiles of 253 patients in the prospective validation cohort
