# Supplementary material for: Proteomics-driven noninvasive screening of circulating serum protein panels for the early diagnosis of hepatocellular carcinoma
Source: Nat Commun. 2023 Dec 18;14:8392. doi: 10.1038/s41467-023-44255-2 (PMC10728065; doi:10.1038/s41467-023-44255-2)
Supplement: Supplementary file 14 — Reporting Summary [file 41467_2023_44255_MOESM14_ESM.pdf]

nature portfolio

Corresponding author(s): Xiaolong Liu

Last updated by author(s): Nov. 14, 2023

Reporting Summary

Nature Portfolio wishes to improve the reproducibility of the work that we publish. This form provides structure for consistency and transparency in reporting. For further information on Nature Portfolio policies, see our Editorial Policies and the Editorial Policy Checklist.

Statistics

For all statistical analyses, confirm that the following items are present in the figure legend, table legend, main text, or Methods section.

n/a	Confirmed	
☐	☒	The exact sample size (*n*) for each experimental group/condition, given as a discrete number and unit of measurement
☐	☒	A statement on whether measurements were taken from distinct samples or whether the same sample was measured repeatedly
☐	☒	The statistical test(s) used AND whether they are one- or two-sided<br>*Only common tests should be described solely by name; describe more complex techniques in the Methods section.*
☒	☐	A description of all covariates tested
☐	☒	A description of any assumptions or corrections, such as tests of normality and adjustment for multiple comparisons
☐	☒	A full description of the statistical parameters including central tendency (e.g. means) or other basic estimates (e.g. regression coefficient) AND variation (e.g. standard deviation) or associated estimates of uncertainty (e.g. confidence intervals)
☐	☒	For null hypothesis testing, the test statistic (e.g. *F*, *t*, *r*) with confidence intervals, effect sizes, degrees of freedom and *P* value noted<br>*Give P values as exact values whenever suitable.*
☒	☐	For Bayesian analysis, information on the choice of priors and Markov chain Monte Carlo settings
☒	☐	For hierarchical and complex designs, identification of the appropriate level for tests and full reporting of outcomes
☐	☒	Estimates of effect sizes (e.g. Cohen's *d*, Pearson's *r*), indicating how they were calculated

Our web collection on statistics for biologists contains articles on many of the points above.

Software and code

Policy information about availability of computer code

Data collection

Spectronaut version. 13.2.19 was used for quantifying raw data from DDA- and DIA-MS. Skyline version 3.6.0.1 was used for quantifying quantitative data from PRM-MS.

Data analysis

Wkomics (https://omicsolution.org/wkomics/main/) analysis platform was used for the pre-processing of the proteomic data, statistical analysis and GO and KEGG analysis. Graphpad PRISM version 8 was used for drawing scatter plot, box plot, violin plot and column of data visualization. Online analysis tool String (https://cn.string-db.org/) was used for protein-protein interaction (PPI) analysis and Cytoscape 3.9.1 was used for the visualization of PPI network. Data analysis was perform in R version 4.0.2. using custom or publicly-available R package. Individual packages are explicitly cited in the manuscript and listed below. The code is available upon request and deposited in a Github repository. We have obtained a DOI (doi.org/10.5281/zenodo.10117967) for the Github repository at Zenodo.

Packages:
randomForest v4.6-14
pROC v1.18.0
reshape2 v1.4.4
corrplot v0.92

For manuscripts utilizing custom algorithms or software that are central to the research but not yet described in published literature, software must be made available to editors and reviewers. We strongly encourage code deposition in a community repository (e.g. GitHub). See the Nature Portfolio guidelines for submitting code & software for further information.

Data

Policy information about availability of data

All manuscripts must include a data availability statement. This statement should provide the following information, where applicable:

- Accession codes, unique identifiers, or web links for publicly available datasets
- A description of any restrictions on data availability
- For clinical datasets or third party data, please ensure that the statement adheres to our policy

The raw data of mass spectrometry generated in this study have been deposited in the PRIDE database51 under accession code PXD046887 (https://www.ebi.ac.uk/pride/archive?keyword=PXD046887). Source data are provided with this paper.

Human research participants

Policy information about studies involving human research participants and Sex and Gender in Research.

Reporting on sex and gender	In the discovery cohort, gender information is available in Supplementary Data 1. Gender based analysis is performed and reported in Supplementary Data 2. In the validation cohort, 345 (80.4%) male and 84 (19.6%) female patients were included. And in the pospective cohort, 205 (81.0%) male and 48 (19.0%) female were included.
Population characteristics	For the discovery cohort involving serum samples, all the clinical characteristics are shown in Supplementary Data 1 and their potential as confounding factors has been assessed in chi-square analysis (Supplementary Data 2).<br>For the validation cohort involving serum samples, no clinical characteristics are obtained.
Recruitment	All the patients who met inclusion criteria in the clinical specimen banks were included. In the prospective cohort, the median follow-up time of 253 LC patients was 43.9 months and 36 LC patients were diagnosed with HCC during subsequent follow-up. The median time from enrollment to progression to HCC for these LC patients was 28.1 months.<br>A description of the inclusion criteria was involving in the manuscript.
Ethics oversight	This project was approved by the Institution Review Board of Mengchao Hepatobiliary Hospital of Fujian Medical University. Informed consent was obtained from each participant before the operation. The use of clinical specimens was completely in compliance with the "Declaration of Helsinki".

Note that full information on the approval of the study protocol must also be provided in the manuscript.

Field-specific reporting

Please select the one below that is the best fit for your research. If you are not sure, read the appropriate sections before making your selection.

☒ Life sciences ☐ Behavioural & social sciences ☐ Ecological, evolutionary & environmental sciences

For a reference copy of the document with all sections, see nature.com/documents/nr-reporting-summary-flat.pdf

Life sciences study design

All studies must disclose on these points even when the disclosure is negative.

Sample size	No sample size calculation was performed. But we estimate the outcome of model groups (at least 10 outcomes), and set the sample size (more than 100 samples) according to the fact that there must be more than 10 samples for each grouping outcome (PMID: 8417638).
Data exclusions	No data were excluded from the analyses.
Replication	No replicated measurement of samples was performed in the study. MS data collection all used independent samples.
Randomization	In random forest model, samples were divided into a training set and a testing set at a ratio of 7: 3 randomly.
Blinding	Investigators were not blind during the analysis. This is not an intervention study for patients, but an analysis of the pathological characteristics of patients under non-intervention measures. We need to collect samples according to the patient 's disease diagnosis. However, we did not artificially screen the patient 's subgroups of pathological characteristics to ensure the authenticity and representativeness of our cohorts.

Reporting for specific materials, systems and methods

We require information from authors about some types of materials, experimental systems and methods used in many studies. Here, indicate whether each material, system or method listed is relevant to your study. If you are not sure if a list item applies to your research, read the appropriate section before selecting a response.

Materials & experimental systems

n/a	Involved in the study
☒	☐ Antibodies
☒	☐ Eukaryotic cell lines
☒	☐ Palaeontology and archaeology
☒	☐ Animals and other organisms
☒	☐ Clinical data
☒	☐ Dual use research of concern

Methods

n/a	Involved in the study
☒	☐ ChIP-seq
☒	☐ Flow cytometry
☒	☐ MRI-based neuroimaging
